# Contrasting intra-individual variation in size-based trophic and habitat shifts for two coastal Arctic fish species

**DOI:** 10.1007/s00442-023-05423-9

**Published:** 2023-07-24

**Authors:** Harri Pettitt-Wade, Nigel E. Hussey, Colin P. Gallagher, Ellen V. Lea, Danielle L. Orrell, Lisa L. Loseto

**Affiliations:** 1 Fisheries and Oceans Canada, Freshwater Institute, Winnipeg, MB R3T 2N6 Canada; 2 Integrative Biology, University of Windsor, Windsor, ON N9B 3P4 Canada; 3 Fisheries and Oceans Canada, Inuvik, NT X0E 0T0 Canada; 4 Environment and Geography, University of Manitoba, Winnipeg, MB R3T 2N2 Canada

**Keywords:** Individual specialization, Stable isotopes, Dietary shift, Greenland cod, Arctic char

## Abstract

Within and among species variation in trophic and habitat shifts with body size can indicate the potential adaptive capacity of species to ecosystem change. In Arctic coastal ecosystems, which experience dramatic seasonal shifts and are undergoing rapid change, quantifying the trophic flexibility of coastal fishes with different migratory tactics has received limited attention. We examined the relationships among body length and condition (Fulton’s K, phase angle from Bioelectrical Impedance Analysis) with trophic and habitat shifts (differences in δ^15^N and δ^13^C between blood tissues with different turnover rates) of two abundant and culturally important species, anadromous Arctic char (*Salvelinus alpinus*, *n* = 38) and sedentary Greenland cod (*Gadus ogac*, *n* = 65) during summer in coastal marine waters near Ulukhaktok, Northwest Territories, Canada. Habitat shifts (δ^13^C) increased with length (i.e., pelagic to benthic-littoral) and crossed-equilibrium (zero) at mid-sizes for both species. Seasonal trophic shifts (δ^15^N) were generally positive (i.e., increasing trophic level) for Arctic char and negative for Greenland cod. As hypothesised, intra-individual variation in size-based trophic shifts (δ^15^N-length residuals) increased with length for Arctic char. However, there were no trends with length in Greenland cod. Our findings highlight the importance of flexibility through ontogeny and mobility for Arctic char, whereas Greenland cod were generalist to localized prey and habitat across all sizes. The significant effect of body condition (phase angle) on size-based trophic shifts in Arctic char, and size-based habitat shifts in Greenland cod, highlight the potential trade-offs of contrasting life history strategies and capacity for ontogenetic niche plasticity.

**Supplementary Information:**

The online version contains supplementary material available at 10.1007/s00442-023-05423-9.

## Introduction

Ecological theory often focusses on the response of populations to ecosystem processes accounting for factors such as age and body size (Werner and Gillium 1984; Fisher et al. 2010), but the potential importance of variation within and among individuals is not commonly considered (Bolnick et al. 2003). Intra-individual variation in phenotypic traits provides the basis for flexible behaviour and can indicate a species’ adaptive capacity to environmental and food web changes (Bolnick et al. 2011). For example, dietary plasticity in Arctic char (*Salvelinus alpinus*) might facilitate coexistence with European whitefish (*Coregonus lavaretus*) in freshwater lakes, where the latter is invasive and competitively superior (Eloranta et al. 2011). In Arctic marine ecosystems, climate change is rapidly shifting the timing of seasonal events by extending the duration of the open water summer season and shortening the ice-covered winter season (Stroeve et al. 2012). These broadscale climate impacts are modifying food web structure and redistributing conventional prey throughout the Arctic (Fossheim et al. 2015). The consequence of these changes for endemic species survival and their adaptive capacity requires detailed knowledge of species’ ecology, which is often lacking for Arctic fishes (Dey et al. 2018), and for shallow coastal marine ecosystems (i.e., < 200 m depth) which are among the most dynamic and anthropogenically impacted of Arctic environments (Jackson et al. 2001; Sheaves 2009).

Seasonal variation in migratory species diversity and abundance is an important feature of coastal marine ecosystems, partly attributed to strong tidal regimes and broad seasonal shifts in prey availability associated with temperature, salinity, light and nutrient dynamics (Sheaves 2009; Steiner et al. 2015). In the Arctic, coastal marine ecosystems undergo pronounced seasonal transitions, whereby, for example, temperatures, light levels, hydrology and turbidity rapidly span a broad range, altering primary nutrient availability in the coastal nearshore from ice algae to terrestrial river or glacial run-off and phytoplankton in the epilimnion (Emmerton et al. 2008; McGovern et al. 2020). Fish species have evolved diverse strategies to benefit from this seasonality, for example, with some species limited to marine presence during the open water summer season (e.g., anadromous Arctic char, Jørgensen and Johnsen 2014), whereas others are able to feed in the coastal marine environment year-round (e.g., Greenland cod *Gadus ogac*, Morin et al. 1991).

Understanding the dietary and habitat flexibility of species with divergent life histories and functional ecologies is central to determining the broad ranging effects of climate change on Arctic coastal marine food webs. Individual flexibility in movement, habitat-use, and diet are primary means available to mobile aquatic predators for responding to changes in prey availability (Dill 1983). Changes in these ecological traits often have consequences for life history parameters. For example, while the ecology of Arctic char during the marine phase is relatively understudied, research has identified considerable intra-individual variation in diet and growth rate leading to resource polymorphisms within and among temperate and sub-Arctic lakes (e.g., Andersson et al. 2005; Knudsen et al. 2007). This observed plasticity suggests an adaptive potential that may buffer the effects of ecosystem change (Andersson et al. 2005; Skúlason et al. 2019). Similarly, Greenland cod, an often-abundant Arctic coastal fish, has received limited attention with most published work undertaken at temperate latitudes (e.g., Morin et al. 1991; Nielsen and Andersen 2001; Knickle and Rose 2014a). Juvenile Greenland cod in west Greenland exhibit density-dependent habitat flexibility (Laurel et al. 2003, 2004), while dietary and habitat flexibility associated with body size occurs among sub-Arctic close relatives Atlantic cod (*Gadus morhua*, Meager et al. 2018), walleye Pollock (*Theragra chalcogramma*) and Pacific cod (*Gadus macrocephalus*, Laurel et al. 2009; Marsh et al. 2012). These findings suggest that Greenland cod may be flexible to changes in prey and habitat availability, but relationships between intra-individual variation in diet and body size remain unclear. Modelled distributions of Arctic char and Greenland cod for the year 2050 based on IPCC RCP8.5 emissions scenario indicate increased probability of occurrence at higher latitudes (aquamaps.org, updated October 2019; Scarponi et al. 2018). Therefore, comparing intra- and inter-individual dietary flexibility among species where they occur in sympatry provides an important tool to predict the impacts of food web restructuring on Arctic marine ecosystems.

The measurement of carbon (δ^13^C) and nitrogen (δ ^15^N) stable isotope values in various fish tissues provides a reliable technique for examining intra- and inter-individual variation in diet and habitat-use (Bearhop et al. 2004; Vander Zanden et al. 2015). Stable isotopes can be used to infer diet and habitat of individuals through δ^15^N indicating trophic level, and δ^13^C varying spatially as a result of basal carbon sources in prey consumed (e.g., between terrestrial, freshwater, and marine: Vander Zanden et al. 1997; benthic and pelagic: e.g., Hobson et al. 1995). Diet derived stable isotopes are assimilated into body tissues at different but predictable rates (i.e., tissue turnover rates: Vander Zanden et al. 2015). As such, comparison of δ^15^N and δ^13^C values in two or more tissues can indicate diet consumed and habitat occupied over different temporal scales (Shipley and Matich 2020).

Here we compare intra-specific variation in size-based seasonal trophic (δ^15^N) and habitat (δ^13^C) shifts over spring–summer in Arctic char and Greenland cod as an indicator of their flexibility and adaptability to a rapidly changing Arctic ecosystem. We examine both inter-individual (single tissue; i.e., trophic averaging) and intra-individual variation (paired-tissue differences; i.e., trophic and habitat shifts) in body length-isotope relationships from fast (days-weeks; plasma) and slow (weeks-months; red blood cells (RBC)) turnover tissues (Vander Zanden et al. 2015). In addition, we measure body condition indices and examine their interactions with variation in size-based trophic and habitat shifts to infer potential drivers or consequences of observed flexibility for each species. We hypothesise that intra-specific variation in seasonal trophic shifts will increase with length for Arctic char and Greenland cod (i.e., greater diet variation among larger fish than smaller fish), representing ‘ontogenetic niche shifts’ (Polis 1984; Fokkema et al. 2020). For diet-related habitat shifts, we hypothesise higher intra-specific variation in size-related shifts in Arctic char due to their seasonal migrations between freshwater and marine environments versus year-round marine foraging by Greenland cod. We predict that intra-specific variation in δ^13^C and δ^15^N will increase with body condition indices, whereby higher diet variation coupled with higher condition will reflect the importance of flexible feeding strategies for fitness during the highly seasonal productivity pulse in the Arctic. To test these hypotheses, we followed the methods of Matich et al. (2019) whereby a series of body length-isotope regressions were used to compare the relative strength of ontogenetic trophic shifts, and post-hoc *t* tests to examine the effect of condition indices on trophic shift-regressions.

## Methods

### Sample site and collection

Fish were collected using a combination of gill nets (114 mm and 140 mm stretch mesh) and angling in the coastal bays of Ulukhaktok, Northwest Territories, Canada (N 70.73444°, W − 117.77652°) and within Safety Channel, Prince Albert Sound (N 70.59251°, W − 117.247537°) on 20-Jul to 12-Aug-2018 (Arctic char) and 12-Jul to 05-Aug-2018 (Greenland cod). Arctic char and Greenland cod were identified as a research priority due to their importance in subsistence fisheries and the coastal marine food web (Lea et al. 2023b). For each fish, we recorded the fork length (Arctic char) or total length (Greenland cod, referred to as body length (BL) for both species from hereon) [mm], wet mass [g], and used a hand-held Bioelectrical Impedance Meter (Certified Quality Reader, Seafood Analytics, CQ Foods, Inc., MI, USA) to measure reactance and resistance indicative of body condition (phase angle; Hartman et al. 2015). Blood samples were taken from the caudal vein using 2 ml heparinized syringes, spun in a centrifuge to separate RBC from plasma and frozen in heparinized Eppendorf vials for later stable isotope analysis. Fishes were photographed and either euthanised for full-body sampling or tagged with internal transmitters and released for separate acoustic telemetry (Hollins et al. 2022) and polymorphism (Burke et al. 2022) projects.

### Body condition indices (Fulton’s K, phase angle)

We examined two measures of condition in the fish: Fulton’s K and phase angle. Standard Fulton’s *K* was calculated using the equation:

$$K=\frac{W\times {10}^{5}}{{L}^{3}}$$

where *W* = wet mass (g) and *L* = body length (mm). Phase angle was estimated using Bioelectrical Impedance Analysis following the methods of Hartman et al. (2015). In brief, a mild electrical current (800 μA AC and 50 kHz) was passed through the fish and the impedance (ratio of resistance and reactance of tissue to applied electrical current; Cox and Heintz 2009) was measured using the Bioelectrical Impedance Meter. Lipids are non-conductive; therefore, higher resistance (*R*) values equate to higher tissue lipid content. Reactance (*X*) is sensitive to cell volume and, therefore, should relate to the volume of total healthy cells within tissues (Hartman et al. 2015). The relationship between reactance and resistance (both measured in ohms (Ω)) was used to determine phase angle, which is proportional to body condition (Cox and Heintz 2009; Hartman et al. 2015), using the equation:

$$Phase\,angle\left(^\circ \right) = {\text{Arctan}} \left( \frac{X}{R} \right) \times \frac{180}{\pi }$$

To obtain resistance and reactance values, each fish was placed left side up on a non-conductive surface and four electrodes within the device pressed against the skin dorsolaterally and compressed to 50% (~ 1 cm above and parallel to the lateral line, 100 mm total distance between electrodes). The electrodes were centred in line with the anterior base of the pectoral fin.

### Stable isotope analysis

Plasma and RBC samples were freeze dried and ground to a homogenous powder using a mortar and pestle. Lipids were extracted using the Solvent Distillation method whereby 2:1 chloroform:methanol solution was added to homogenised samples, agitated and left in a 30 °C water bath for 24 h. The solvent was then decanted, and samples air-dried in a fume hood. Samples and standards were then weighed into tin cups (5 mm × 9 mm) and isotope values measured on an isotope ratio mass spectrometer (IRMS, Biotracers Lab; Fisheries and Oceans Canada’s Freshwater Institute, Manitoba, Canada) coupled with an elemental analyser (Lab. Costech-4010 EA). Carbon and nitrogen relative abundances are expressed in delta notation ($\delta R$) using the following equation:

$$\delta R\, \left( \permille \right) = \left( {\frac{{R_{{{\text{sample}}}} }}{{R_{{{\text{standard}}}} }} - 1} \right) \times 1000$$

where R refers to the ratio of either ^15^N/^14^N or ^13^C/^12^C within samples and standards. Sample data was compared with standard reference materials for CO_2_ (Vienna Pee Dee Belemnite) and N_2_ (atmospheric nitrogen). Standards and triplicates were included on each run. Repeats of in-house fish muscle standard had a standard deviation of < 0.05 and < 0.1 for δ^13^C and δ^15^N, respectively. Standard deviation precision of USGS standards from 60 repeats were < 0.08 (USGS40, δ^13^C) and < 0.12 (USGS41a, δ^15^N).

### Differences in δ^13^C and δ^15^N between tissues

Tissue-matched isotope data (body length, plasma and RBC) were obtained for 31 Arctic char and 48 Greenland cod (out of 38 and 65 total, respectively). Note slightly larger sample sizes for single tissue data (38 and 64 RBC, 31 and 49 plasma, Arctic char and Greenland cod, respectively). Prior to calculating differences in δ^13^C and δ^15^N between fast (days-weeks; plasma) and slow (weeks-months; RBC) turnover tissues for each individual, a diet-tissue correction (DTC) was applied to account for differences in tissue-specific turnover (i.e., half-life) between tissues. Following Matich et al. (2019), raw (directional; i.e., demonstrating isotope enrichment or depletion) and absolute (unidirectional; i.e., total difference) tissue differences in δ^13^C and δ^15^N (*δR*_*tissdiff*_) were calculated for each individual by subtracting the RBC value from DTC plasma value:

$$\delta R_{tissdiff} = \left( {\delta R_{plasma} + \ln \left( {half{ - }life} \right)} \right) - \delta R_{RBC}$$

where *R* refers to δ^13^C or δ^15^N and ln(*half-life*) refers to the estimated isotopic half-life (i.e., turnover rate) for plasma in each individual. Body mass half-life relationships were derived from a synthesis of experimental values for vertebrate ectotherms and plasma half-life calculated for each individual using the following equation: ln(*half-life*) = 0.21 × ln(*body mass*) + 2.47 (Eq. 3 in Vander Zanden et al. 2015). All paired tissue differences refer to data with the DTC applied.

### Statistical analyses

All variables (body length, condition indices, and δ^13^C and δ^15^N values of RBC and plasma for both Arctic char and Greenland cod) showed equal variance and generally fitted a normal distribution. Extreme outliers (> 3 SD) were removed prior to analyses (data for three Arctic char and two Greenland cod). Species differences in stable isotope values (δ^13^C, δ^15^N, and tissue differences), body length, and condition indices (Fulton’s K, phase angle) were first examined using Welch’s *t*-tests. Following the methods of Matich et al. (2019), a series of least squares regressions were then used to estimate different aspects of inter- and intraspecific variation in ontogenetic trophic shifts for the two species (Table 1, Online Resource 1):

1. To estimate the strength and direction of variation over time derived from habitat (δ^13^C) versus trophic (δ^15^N) factors, regressions were conducted on raw δ^13^C and δ^15^N between plasma (days-weeks) and RBC (weeks-months).
2. To estimate the relationships between length and inter-individual variation in habitat versus trophic factors at the two time periods (i.e., single tissue, trophic averaging), regressions were conducted for each tissue on δ^13^C and δ^15^N with body length.
3. To estimate relationships between body length and intra-individual habitat or trophic shifts (i.e., paired-tissue differences), regressions were conducted between length and tissue differences in δ^13^C and δ^15^N (directional).
4. To estimate the relationships between body length and inter-individual variation in habitat or trophic factors for the two time periods, the absolute (unidirectional) residuals derived from Analysis step 2 (length vs. δ^13^C or δ^15^N) were regressed against length for each tissue.
5. The relationships between body length and intra-individual variation in habitat or trophic shifts were examined by regressing the absolute residuals derived from Analysis step 3 (length vs. tissue difference in δ^13^C or δ^15^N) against length.

**Table 1:** Parameters included and specific aims for least squares regressions conducted to examine inter and intra-individual variation in ontogenetic trophic and habitat shifts in Arctic char and Greenland cod following the analytical framework of Matich et al. (2019)

^Step^	Predictors	Dependent variables^A^	Derived variables^A^	Aim	Resource
1	δ^13^C_plasma_	δ^13^C_RBC_		Estimate strength and direction of variation over days to weeks derived from habitat versus trophic factors	Online Resource 2
	δ^15^N_plasma_	δ^15^N_RBC_			
2	BL	δ^13^C_plasma_	a. BL vs. δ^13^C_plasma_ abs residuals	Estimate the relationships between length and inter-individual variation in habitat versus trophic factors at the two time periods (i.e., single tissue, trophic averaging)	Figure 1a, b
		δ^13^C_RBC_	b. BL vs. δ^13^C_RBC_ abs residuals		
		δ^15^N_plasma_	c. BL vs. δ^15^N_plasma_ abs residuals		Figure 1c, d
		δ^15^N_RBC_	d. BL vs. δ^15^N_RBC_ abs residuals		
3	BL	δ^13^C_diff_	e. BL vs. δ^13^C_diff_ abs residuals	Estimate relationships between body length and intra-individual in habitat or trophic shifts (i.e., paired tissue differences)	Figure 2a, b
		δ^15^N_diff_	f. BL vs. δ^15^N_diff_ abs residuals		Figure 2c, d
4	BL	a		Estimate relationships between body length and inter-individual variation in habitat or trophic factors for the two time periods	Figure 3a, b
		b			
		c			Figure 3c, d
		d			
5	BL	e		Examine intra-individual variation in ontogenetic trophic shifts	Figure 4a, b
		f			Figure 4c, d

See Table 2 for parameters used in Welch’s *t* test species comparisons, and Table 3 for post-hoc *t*-tests of the effect of body condition indices on ontogenetic trophic shifts

*BL* Body length (fork length (Arctic char), total length (Greenland cod), mm). *abs* absolute (unidirectional). *diff* tissue difference from isotope value of RBC subtracted from discrimination-corrected isotope value of plasma

^A^Each row represents a different regression

^a–f^Lower-case lettering denotes the derived residuals that were used in regressions 4–5

^Step^Scale qualifier for the sequence of regressions

Regressions were conducted using both single-tissue (Analysis steps 1, 2, 4) and paired-tissue differences (Steps 3, 5) given the potential varied insight to be gained from each approach depending on the species examined (Matich et al. 2019). Specifically, these regressions indicate whether each species exhibited benthic or littoral feeding relative to pelagic (δ^13^C), or higher or lower trophic level diets (δ^15^N, step 1), and trends with body size representing changes in these factors through ontogeny (Steps 2–5). Given the different turnover rates of the two tissues and relationships between body length and life history, regressing calculated tissue-differences with body length indicates the speed and direction of ontogenetic trophic shifts within (Step 3) and among individuals (Step 2). Size-based increases or decreases in variation in trophic shifts should be reflected in likewise increases or decreases in the range of residuals from the regression models. As such, regressing residuals from size-based trophic shift regressions with length should indicate whether inter (Step 4) or intra- (Step 5) individual variation in ontogenetic trophic shifts change with body size. As per Matich et al. (2019), quadratic regressions were chosen over linear regressions when there were large improvements of *R*^2^ and *F* values. Post hoc *t* tests were then used to examine the effects of condition indices on ontogenetic trophic shifts given the often-important relationships between diet, body condition, and ontogeny.

All statistical analyses were performed in IBM SPSS Statistics (v.26) with *α* = 0.05.

## Results

Arctic char and Greenland cod shared similar ranges in body size (265 mm, 305 mm, respectively) and Fulton’s K (total mass body condition, Table 2), indicating comparable capacity for trophic variation associated with range in size and condition. Arctic char were notably larger (mean ± SE body length, 754 ± 12 (*n* = 38) and 441 ± 7 mm (*n* = 65), Arctic char and Greenland cod respectively, all Welch’s *t* test, *P* < 0.05), and Greenland cod showed larger variation in Fulton’s K (SE = 1.12 compared to 0.77 in Arctic char, Table 2). In contrast, phase angle was higher in Greenland cod compared to Arctic char (73.29 ± 0.37 ω and 66.72 ± 0.46 ω, respectively, *P* < 0.05). Higher phase angle indicates lower lipid content and higher body condition due to greater cell density and intact cell membranes within tissues, which allows for more efficient transfer of energy within tissues (Cox and Heintz 2009; Hartman et al. 2015).

**Table 2:** Arctic char and Greenland cod body length, condition indices, red blood cell (RBC) and plasma δ^13^C and δ^15^N values, and paired tissue differences

	Arctic char					Greenland cod				
	*n*	Mean ± SE	Range	Min	Max	*n*	Mean ± SE	Range	Min	Max
Length (mm)^1^	38	754 ± 12*	265	635	900	65	441 ± 7	305	280	585
Mass (kg)^2^	37	4.49 ± 0.15*	3.40	2.70	6.10	59	0.97 ± 0.05	1.70	0.30	2.00
Fulton’s K^3^	37	1.07 ± 0.03	0.77	0.63	1.40	59	1.11 ± 0.03	1.12	0.67	1.79
Phase angle^4^	36	66.7 ± 0.46	13.8	59.8	73.6	47	73.3 ± 0.37*	15.2	66.1	81.3
δ^13^C Plasma	31	− 22.0 ± 0.10	2.13	− 23.0	− 20.8	49	− 19.8 ± 0.13*	3.97	− 21.6	− 17.6
δ^15^N Plasma	31	16.8 ± 0.12	3.11	15.3	18.4	49	16.6 ± 0.11	3.08	15.2	18.3
δ^13^C RBC	38	− 22.6 ± 0.09	2.61	− 23.9	− 21.3	64	− 20.6 ± 0.08*	3.17	− 21.9	− 18.8
δ^15^N RBC	38	15.3 ± 0.10	3.20	14.1	17.3	64	16.6 ± 0.11*	4.15	14.8	18.9
δ^13^C diff^5^	31	− 0.11 ± 0.10	2.90	− 1.32	1.58	48	0.02 ± 0.08	2.37	− 1.09	1.28
δ^15^N diff^5^	31	0.79 ± 0.13*	3.05	− 0.49	2.56	48	− 0.68 ± 0.10	3.45	− 2.31	1.14
δ^13^C diff abs^6^	31	0.42 ± 0.07	1.55	0.03	1.58	48	0.40 ± 0.05	1.28	0.00	1.28
δ^15^N diff abs^6^	31	0.84 ± 0.12	2.52	0.04	2.56	48	0.84 ± 0.06	2.29	0.02	2.31

*Significantly higher than the second species (Welch’s *t* tests, *P* < 0.05)

^1^Fork length (Arctic char), total length (Greenland cod)

^2^Wet body mass

^3^Body condition from cubed length–weight relationship

^4^Body condition from the relationship between whole fish reactance and resistance, indicative of tissue condition based on spacing between tissue cells

^5^Paired tissue difference in δ^13^C or δ^15^N from subtraction of slow turnover tissue data (red blood cell) from discrimination-corrected fast turnover tissue data (plasma)

^6^Paired tissue differences converted to unidirectional absolute values

### Interspecific differences in plasma and RBC δ^13^C and δ^15^N

Interspecific comparisons of plasma and RBC δ^13^C indicated that, overall, Greenland cod exhibited more benthic/littoral feeding and over a greater range of habitats than Arctic char (Online Resource 1, Table 1 step 1). Specifically, ^13^C was more enriched and had a greater range in Greenland cod compared to Arctic char in both plasma (all Welch’s *t* test, *t*_1,78_ = 147, *P* < 0.001) and RBC (*t*_1,100_ = 233, *P* < 0.001, Table 2). Interspecific comparison of RBC δ^15^N indicated that Greenland cod fed at a higher trophic level over the previous weeks to months compared to Arctic char (*t*_1,100_ = 59.3, *P* < 0.001), whereas no significant difference in plasma δ^15^N indicated that the species fed at similar trophic levels over the previous days to weeks (*t*_1,78_ = 0.83, *P* = 0.36).

### Interspecific comparison of paired-tissue differences for δ^13^C and δ^15^N

Comparison of paired directional (i.e., ‘DTC Plasma—RBC’) and absolute (i.e., total difference, unidirectional) δ^13^C-tissue differences indicated that, overall, the frequency and speed of habitat shifts were similar between species, though Greenland cod had a higher range (Table 2). In contrast, comparison of δ^15^N-tissue differences indicated that Arctic char diets generally increased in trophic level over time (0.84 ± 0.15 ‰), whereas Greenland cod diets generally decreased (− 0.62 ± 0.11 ‰, *t*_1,77_ = 84.3, *P* < 0.001) and covered a smaller range (Table 2).

### Relationships between body length and δ^13^C or δ^15^N

**Fig. 1:**
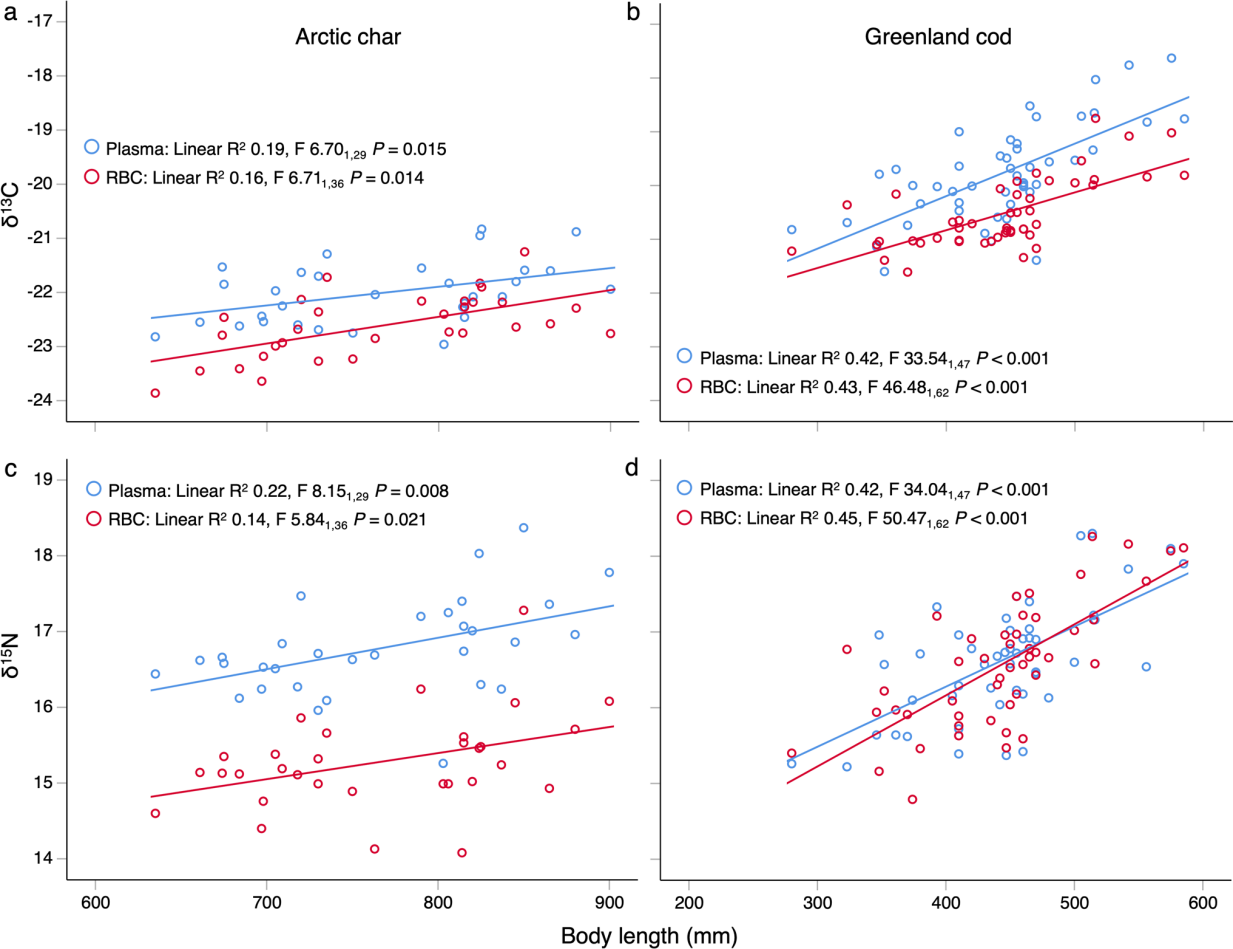
Linear regressions of body length with **a**, **b** δ^13^C and **c**, **d** δ^15^N in plasma and red blood cells (RBC) for Arctic char *(Salvelinus alpinus*) and Greenland cod (*Gadus ogac*). Findings indicated that both species fed more in benthic or littoral (relative to pelagic) habitats and at a higher trophic level with increasing body size. Sample sizes are provided in the F ratio subscripts

Positive linear regressions of body length vs. δ^13^C and δ^15^N indicated that benthic or littoral feeding (relative to pelagic) and prey trophic level generally increased with body size for both species (Fig. 1, Table 1 step 2).

### Relationships between body length and paired-tissue differences (δ^13^C or δ^15^N)

**Fig. 2:**
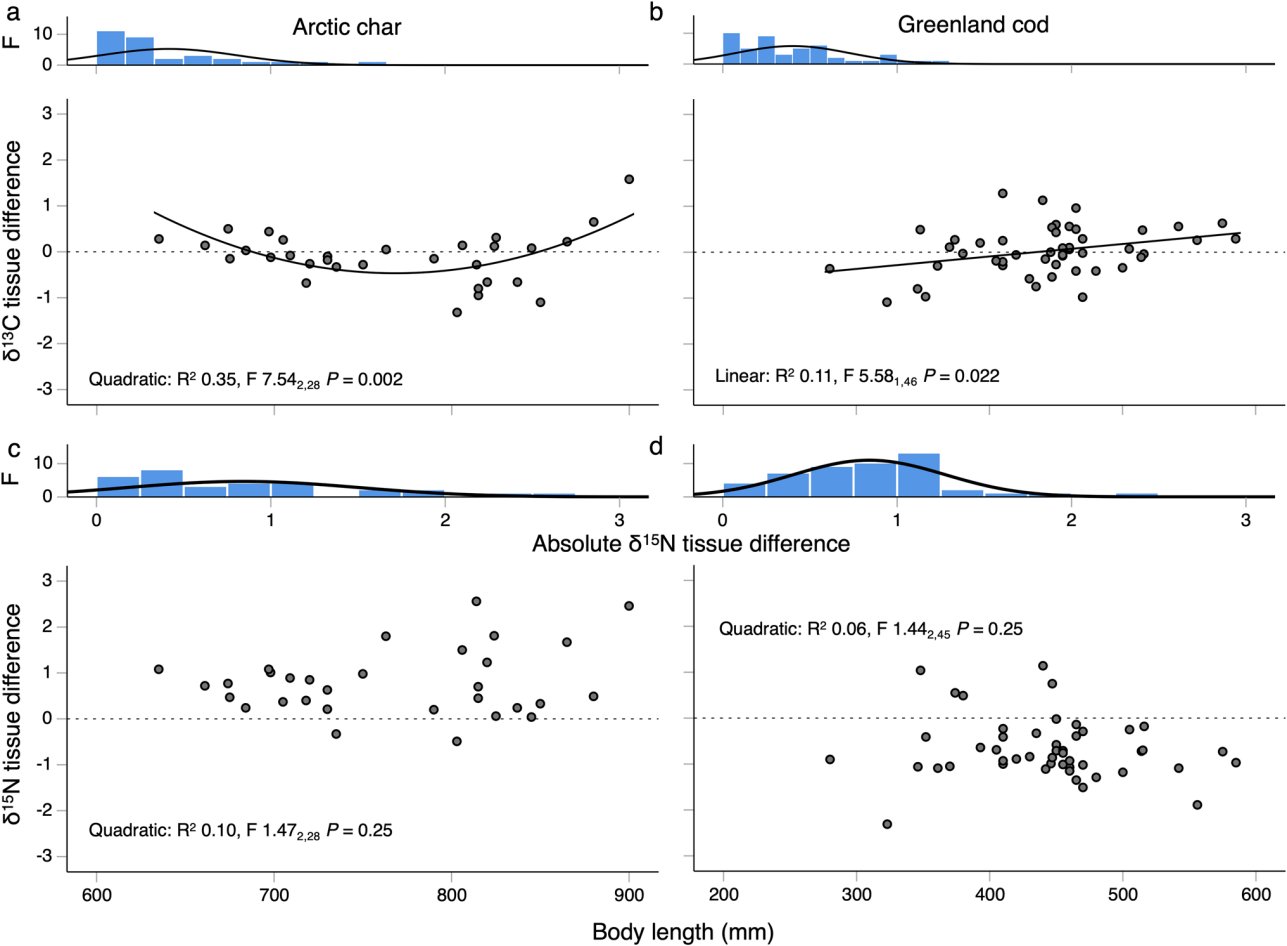
Regressions of body length and diet-tissue corrected differences in **a**, **b** δ^13^C and **c**, **d** δ^15^N between plasma and red blood cells (RBC) for Arctic char and Greenland cod representing habitat and trophic shifts over spring–summer. Frequency histograms (F) are shown for absolute differences. Findings indicated mixed directions in habitat shifts over spring–summer, with a quadratic relationship with body length in Arctic char. A positive linear relationship with body length in Greenland cod indicated a size-based increase in benthic or littoral feeding over spring–summer. Regardless of body length, the majority of Arctic char shifted towards feeding on prey at higher trophic levels, whereas the majority of Greenland cod shifted towards lower trophic prey

Body length and δ^13^C paired-tissue difference regressions signified the prevalence of size-based habitat shifts over spring–summer in both species (Table 1 step 3). For Arctic char, regressions of length vs. δ^13^C-tissue differences exhibited a quadratic relationship (*R*^2^ = 0.35, *F*_2, 28_ = 7.54, *P* = 0.002), whereby δ^13^C-tissue differences were enriched at ~ 650–700 mm, depleted at ~ 700–810 mm, then enriched again from >  ~ 820 mm (Fig. 2a, note low sample size > 850 mm). For Greenland cod, the regression had a positive linear relationship (*R*^2^ = 0.11, *F*_1, 46_ = 5.58, *P* = 0.022, Fig. 2), reflecting a change from negative (δ^13^C depletion, more pelagic) to positive (δ^13^C enrichment, more benthic or littoral) with increasing length (Fig. 2b). Cod approached equilibrium in δ^13^C (i.e., ~ 0 difference) at ~ 350–450 mm in length, despite several individuals at this size having the highest δ^13^C enrichment.

In contrast to δ^13^C, body length and δ^15^N paired-tissue difference regressions indicated that the direction of trophic shifts was similar across body sizes with no clear size-based relationship in either species (Fig. 2c and d). For Arctic char, all but two individuals had tissue differences that reflected δ^15^N enrichment over time (i.e., increasing trophic level; ~ 750 mm: 0–1, >  ~ 750 mm: 0- ~ 2.5). Whereas for Greenland cod, the majority of individuals had tissue differences that reflected a shift to feed on lower trophic level prey in recent days (− 0.68 ± 0.10 ‰ δ^15^N change over time; Fig. 2d).

### Inter-individual variation in size-based trophic and habitat averaging

**Fig. 3:**
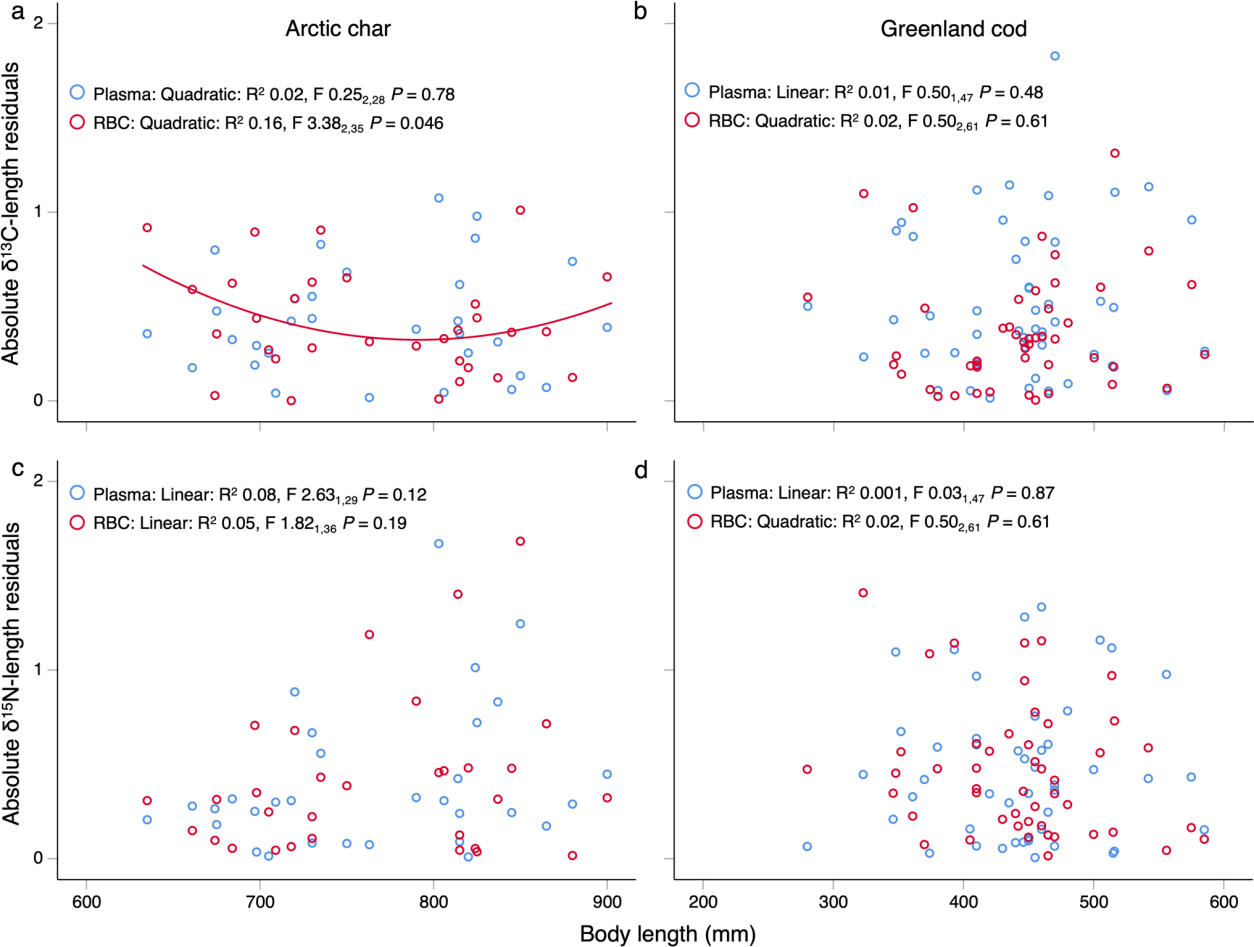
Regressions of body length and absolute **a**, **b** δ^13^C and **c**, **d** δ^15^N body length residuals for Arctic char and Greenland cod plasma and red blood cells (RBC) representing inter-individual variation in size-based habitat and trophic shifts. Findings indicated that inter-individual variation in size-based habitat and trophic shifts do not have a clear relationship with body length in either species

Single-tissue regressions of δ^13^C absolute residuals with length indicated that inter-individual variation in habitat use over weeks to months had a varied relationship for Arctic char depending on body size (Table 1 step 4). Arctic char δ^13^C absolute residuals ranged from 0 to 1 for both tissues, and exhibited a quadratic relationship with length and RBC (*R*^2^ = 0.16, *F*_2, 35_ = 3.38, *P* = 0.046). The regression slope for RBC indicated a decrease in inter-individual variation with length from ~ 1 at ~ 650 mm to ~ 0.4 at ~ 750 mm, then a slight increase to ~ 0.75 at ~ 850–900 mm, though the latter was driven by a small number of individuals (Fig. 3a). Greenland cod demonstrated no clear trends in absolute δ^13^C residuals with length for single tissues, signifying inconsistent inter-individual variation in habitat use relative to body size (Fig. 3b and d). Inter-individual variation in δ^13^C ranged from 0 to ~ 1.5 across both tissues, with the exception of plasma in one ~ 475 mm individual (residuals closer to ~ 2; Fig. 3b).

Single tissue analysis of δ^15^N absolute residuals indicated that body size (or single-tissue analysis) was not a good predictor of inter-individual variation in absolute size-based differences in trophic level (i.e., no clear relationships with length for either species; Fig. 3c and d). Residual values were highest among larger Arctic char (~ 800–900 mm), and mean size Greenland cod (350–450 mm), showing high divergence from body length-isotope relationships for these individuals regardless of time (i.e., tissue).

### Intra-individual variation in size-based trophic and habitat shifts

**Fig. 4:**
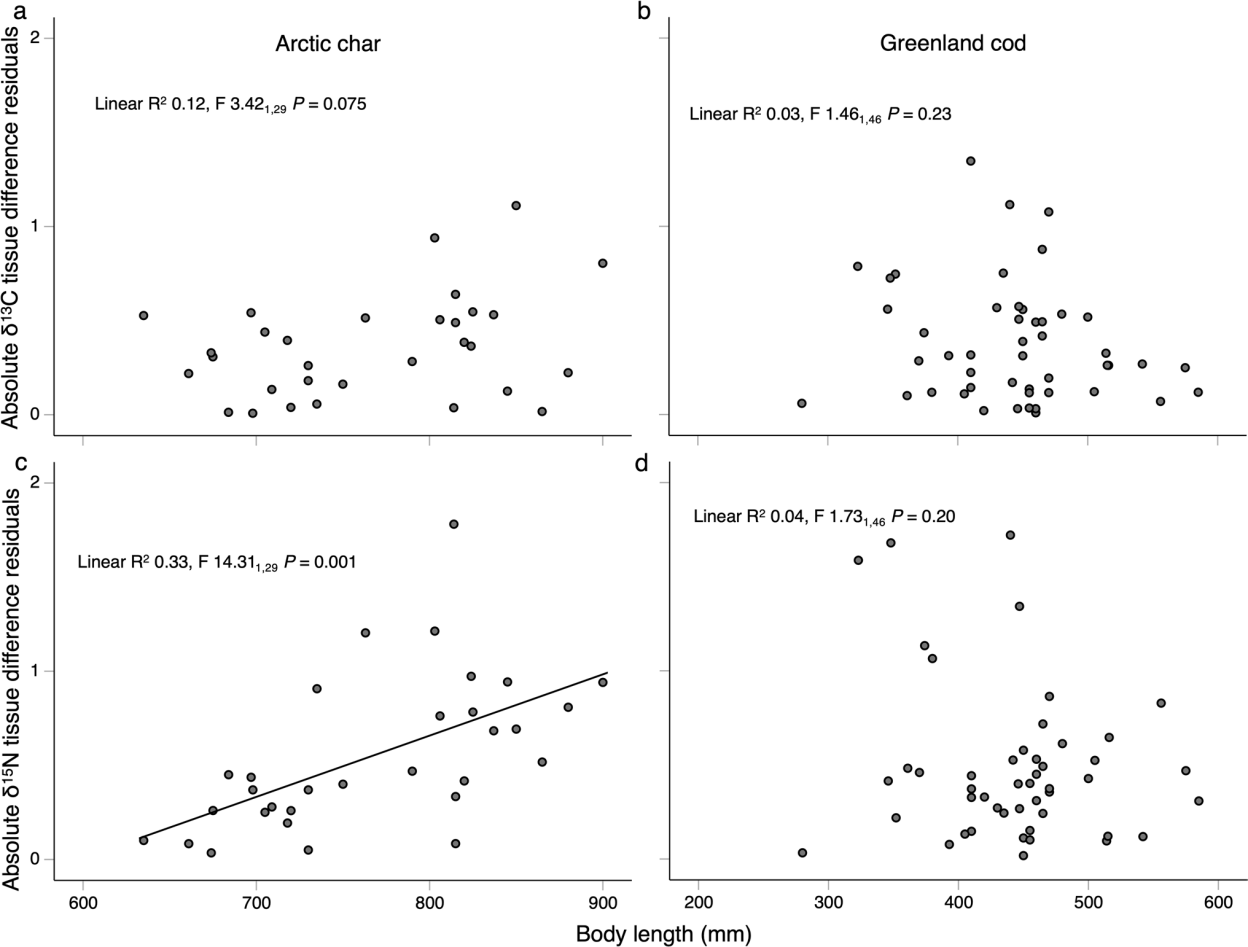
Regressions of body length and absolute **a**, **b** δ^13^C and **c**, **d** δ^15^N diet-tissue corrected tissue difference body length residuals for Arctic char and Greenland cod representing intra-individual variation in size-based habitat and trophic shifts. Findings indicated that intra-individual variation in trophic shifts increases with size in Arctic char, whereas trends were less clear for habitat shifts and not apparent in Greenland cod for trophic or habitat shifts

Evidence suggested that intra-individual variation in trophic shifts increased with length for Arctic char, but the relationship was less clear for habitat shifts (Table 1 step 5). Arctic char δ^15^N paired-tissue difference (absolute residuals, i.e., intra-individual variation in size-based trophic shifts) exhibited a positive linear relationship with length (Fig. 4c, *R*^2^ = 0.33, *F*_1,29_ = 14.3, *P* = 0.001), whereby intra-individual variation in trophic shifts ranged 0 to 1 at <  ~ 750 mm, and ~ 0 to 2 at > 750 mm. The relationship was unclear for intra-individual variation in habitat shifts (δ^13^C) with length in Arctic char based on the quadratic residuals (*R*^2^ = 0.12, *F*_1,29_ = 3.42, *P* = 0.075, Fig. 4a), however, note that linear residuals had a positive linear relationship with length (*R*^2^ = 0.27, *F*_1,29_ = 10.6, *P* = 0.003).

For Greenland cod, the majority of residuals representing intra-individual variation in trophic and habitat shifts were < 1 and none had a clear trend with length (Fig. 4b and d). Greatest absolute residuals for both δ^13^C and δ^15^N were among mean body length (~ 1.8 maximum absolute residual, ~ 400 mm).

### The effect of body condition indices on trophic- and habitat-body length regressions

Post hoc *t*-tests revealed the potential effects of body condition (phase angle) on inter-individual variation in size-based habitat shifts in Greenland cod, and intra-individual variation in size-based trophic shifts in Arctic char. Phase angle had a significant effect on both RBC and plasma single-tissue δ^13^C-length residuals in Greenland cod, and δ^15^N tissue difference-length residuals for Arctic char (Table 3). Regression models were stronger with the addition of phase angle in both cases (Online Resource 3). Julian day of capture had a positive linear relationship with δ^15^N-tissue difference for Arctic char (*R*^2^ = 0.44, *F*_1,29_ = 6.96, *P* = 0.013), but no clear effect on body length relationships or for δ^13^C (i.e., *P* > 0.05 for all).

**Table 3:** Results of post hoc *t*-tests examining the effect of body condition indices on δ^13^C, δ^15^N, paired-tissue differences, and residuals of regressions with body length

	RBC	Plasma	RBC-Plasma^3^	Residual (RBC)^4^	Residual (Plasma)^4^	Residual (RBC-Plasma)^3,4^
Arctic char						
Fulton’s K^1^						
δ^13^C	− 1.38, 0.18	− 1.10, 0.28	− 1.22, 0.24	− 1.92, 0.06	− 1.06, 0.30	0.20, 0.84
δ^15^N	− 0.29, 0.77	− 1.58, 0.13	− 1.15, 0.26	− 0.33, 0.74	− 0.36, 0.72	0.64, 0.52
Phase angle^2^						
δ^13^C	0.10, 0.92	0.50, 0.62	0.12, 0.91	1.40, 0.17	0.99, 0.33	− 0.24, 0.81
δ^15^N	− 0.89, 0.38	0.53, 0.60	1.48, 0.15	0.19, 0.85	1.16, 0.26	**2.32, 0.03**
Greenland cod						
Fulton’s K^1^						
δ^13^C	0.16, 0.87	0.10, 0.92	− 0.42, 0.68	− 1.32, 0.19	− 1.58, 0.12	− 0.77, 0.46
δ^15^N	0.22, 0.83	− 0.37, 0.71	− 0.16, 0.87	0.35, 0.73	0.23, 0.82	− 0.08, 0.94
Phase angle^2^						
δ^13^C	− 0.10, 0.36	0.22, 0.83	− 0.61, 0.55	**2.69, 0.01**	**2.30, 0.03**	0.10, 0.92
δ^15^N	− 0.70, 0.49	− 0.74, 0.47	0.42, 0.68	1.26, 0.21	− 0.76, 0.45	− 1.73, 0.09

Values presented are *t* values and *P* values, respectively. Values in bold are significant at *α* = 0.05. Quadratic regressions (highlighted grey) were chosen over linear regressions when F and *R*^2^ values improved significantly. Body length (mm) refers to fork length (Arctic char) or total length (Greenland cod) (mm)

^1^Body condition from cubed length–weight relationship, therefore, note this is correlated with body length

^2^Body condition from the relationship between whole fish reactance and resistance, indicative of tissue condition based on spacing between tissue cells

^3^Diet-tissue corrections were applied prior to calculating tissue differences

^4^Residuals were converted to unidirectional absolute values for residual regressions

## Discussion

Contrasting levels of intraspecific variation in trophic and habitat shifts with body length were identified for two fish species with different migratory tactics where they co-occur on Arctic marine coasts during summer. Anadromous Arctic char exhibited intraspecific variation in the rate of trophic (δ^15^N) and habitat (δ^13^C) shifts indicating ontogenetic niche flexibility through mobility. In contrast, lack of trends in intraspecific variation with length in Greenland cod indicated that ontogeny is less critical for niche flexibility for this sedentary extreme generalist. Our results indicate distinct evolved strategies to localized conditions among these co-occurring species, which could facilitate coexistence during the spring–summer productivity pulse. Divergent interspecific trends with condition indices highlighted the potential trade-offs of different life-history strategies, and how different functional groups may respond to changes in prey and habitat availability.

Mirroring our findings, the speed and direction of size-based trophic and habitat shifts can vary considerably among temperate-tropical fish species (Bolnick et al. 2003; Shipley and Matich 2020). Using the same method based on split blood isotopes, Matich et al. (2019) found intraspecific variation in ontogenetic trophic shifts varied among coastal and pelagic sharks, and noted the importance of ecosystem stability and complexity for explaining these trends. Our findings provide further evidence for the benefits of examining intraspecific variation in ontogenetic trophic shifts using paired tissue-difference residuals, relative to single-tissue analysis for Arctic teleosts. Similar to Matich et al. (2019), our contrasting results for these two species could also be due to species-specific adaptation to varied ecosystem stability and complexity, although data deficiencies remain on ecosystem complexity for the current study conducted on a remote Arctic marine coast.

### Intraspecific variation in size-based trophic shifts

Our contrasting findings for Greenland cod and Arctic char, despite occurring in some of the same marine environments during summer, likely relates to divergent strategies when responding to seasonal pulses in prey availability (Spares et al. 2012). Ontogenetic trophic shifts may occur for opportunistic generalist species, such as anadromous Arctic char, that exhibit a seasonal migration to exploit prey pulses resulting in rapid growth (Fokkema et al. 2020). This pattern could drive increased intraspecific variation in trophic shifts with body size. Although intraspecific variation in trophic shifts has rarely been investigated among anadromous individuals during their marine migration, Arctic char are often highly plastic with regards to feeding ecology and morphology, and ontogenetic trophic shifts are common among non-migratory lacustrine populations (e.g., Andersson et al. 2005; Guiguer et al. 2005; Kahilainen et al. 2016). Moreover, our finding of a significant trend between body condition (phase angle) and size-based trophic shifts underscore the potential benefit Arctic char gains in nutrition from physiologically costly movements through ontogeny (Cox and Heintz 2009).

When considering Greenland cod, a generalist feeding strategy tied with sedentary year-round residency in dynamic coastal habitats across the full-size range of this species (e.g., Mikhail and Welch 1983; Knickle and Rose 2014a, b) could explain the lack of size-based increase in intraspecific variation in trophic and habitat shifts. For example, Brewster et al. (2018), found Greenland cod from Ulukhaktok and Sachs Harbour (Northwest Territories, Canada) had the highest isotopic niche breadth (ellipses of δ^13^C and δ^15^N) among Arctic gadids. With a diet that often includes benthic macro-crustaceans (e.g., Mikhail and Welch 1989), Greenland cod likely has greater access to prey year-round than Arctic char.

### Importance of habitat for trophic shifts

Size-based dietary habitat shifts often occur in fishes, given that larger individuals possess larger gape size to feed on larger prey, have greater mobility facilitating evasion from predators and can take advantage of ephemeral pulses in prey that require opportunistic and well-timed movements (Fokkema et al. 2020). Stronger trends for habitat- (δ^13^C) than trophic-related (δ^15^N) shifts in Arctic char suggest that the magnitude and direction of the trophic shift are strongly tied to changing foraging locations. The observed quadratic relationship in size-based habitat shifts for Arctic char could be due to sampling a mixture of individuals that originated from different overwintering lakes. During summer, multiple stocks occupy the coastal area sampled (Hollins et al. 2022; Lea et al. 2023a) and different sites of marine entry could vary in base carbon signature, prey availability, ice-off date and migration pathway (Hammer et al. 2021, 2022), altering the time and energy individuals have for marine foraging (Moore et al. 2016; Harris et al. 2020; Hammer et al. 2021). Moreover, three polymorphs were identified in the same Arctic char that were sampled along the coast as the current study using geometric morphometric analysis and acoustic telemetry revealed that the morphotypes did not distinguish between overwintering lakes, highlighting the potential specialization that occurs within the same lake or marine coast (Burke et al. 2022). Given the likelihood of mixed stock individuals (Hollins et al. 2022), polymorphisms (Burke et al. 2022), and associated complex variation in ontogenetic habitat shifts (current study) during coastal marine migration, sampling across body size extremes may benefit future studies in identifying size-based trends.

The lack of clear trends for intraspecific variation in size-based trophic shifts for Greenland cod, together with a positive size-based habitat shift, indicates that ontogenetic shifts in foraging location could have occurred without a change in prey trophic level. The higher δ^13^C in both tissues for Greenland cod was likely due to consumption of more benthic prey (Nielsen and Andersen 2001) versus depleted values for Arctic char indicative of a more pelagic diet (Hansen et al. 2012). Although δ^13^C for both species were within ranges previously reported from muscle tissue (Greenland cod: Brewster et al. 2018; Arctic char: Yurkowski et al. 2018; Ulrich and Tallman 2021), several Greenland cod had low δ^13^C values in the slow turnover tissue, and values were markedly higher in the fast turnover tissue. This contrasting finding suggests a change from feeding on more pelagic or estuarine prey in the previous weeks to more benthic or marine prey over recent days. Morin et al. (1991) identified seasonal shifts in Greenland cod stomach contents (Hudson Bay, Nunavut) from predominantly polychaetes and fish in winter to amphipods in summer that were thought to coincide with changes in temperature and prey abundance. Given that RBC has a maximum turnover rate of several weeks, the trophic shift observed across all body sizes could be due to increasing local availability of lower trophic level prey during and immediately after spring ice-breakup (Hansen et al. 2012). The dichotomous shift for some individuals could be due to partial feeding migrations of specialists within a generalist population, similar to coastal Atlantic cod (Meager et al. 2018), and Greenland cod over small scales at lower latitudes (Morin et al. 1991; Westrheim 1996; Shapiera et al. 2014). The positive effect of phase angle on δ^13^C-body size regressions (both plasma and RBC) in Greenland cod, and δ^15^N-tissue differences in Arctic char suggests that ontogenetic habitat shifts in Greenland cod and trophic shifts in Arctic char may be important for maintaining body condition at larger sizes.

### Life history, maturity and body condition

Seasonal interspecific mismatches in spawning (when fasting is likely) and postspawning recovery may influence the nutritional requirements, body condition, and stable isotope composition at different body lengths during summer. However, body conditions indices were relatively similar and high between the species, with minimal effect on regressions in post hoc tests. Arctic char spawn during fall while overwintering in lakes (Harwood et al. 2013; Jørgensen and Johnsen 2014), whereas Greenland cod spawn during late-winter to early-spring (e.g., Hudson Bay—Morin et al. 1991). The majority of Arctic char (~ > 550 mm or 8 years; Lea et al. 2023a) and Greenland cod (~ > 250 mm or 3 years: Mikhail and Welch 1989) in our study were within the maturity size range previously reported, but size-at-maturity spans a broad range and could not be confirmed for either species. Incorporating more of the smaller immature size-classes in our sample of both species may result in stronger size-based relationships when considering intra-individual variation. Smaller individuals were rarely captured suggesting they may be elsewhere, such as in shallow coastal bays (Greenland cod) or remained in lakes (Arctic char). However, sampling with a smaller gill net mesh and angling hooks would be required to verify the absence of smaller individuals at the study site.

### Species and life-stage specific trophic and habitat preferences

Species and life-stage specific habitat preferences may allow foraging on the same ephemeral prey and in the same geographical location while minimising competition. Benthic habitat associations of Greenland cod in both western Greenland (Nielsen and Andersen 2001) and Newfoundland (Knickle and Rose 2014a) minimised competition with Atlantic cod, which fed more pelagically. Our findings indicated that by mid-July to August, larger (~ > 800 mm) Arctic char switched to feeding on higher trophic level prey, whereas larger (~ > 500 mm) Greenland cod switched to feed on more benthic or littoral prey. These findings are in agreement with previous studies that reported ontogenetic trophic shifts from primarily zooplankton to fish in anadromous (stomach contents: Rikardsen et al. 2000; Dempson et al. 2002) and lacustrine Arctic char (δ^15^N paired tissue differences: McCarthy et al. 2004), and to benthic crustacea in Greenland cod (stomach contents: Morin et al. 1991; Nielsen and Andersen 2001). However, dietary shifts in lacustrine Arctic char have also been reported from benthic prey to zooplankton over summer (including δ^15^N and δ^13^C paired tissue differences: Kahilainen et al. 2016). Although both species feed on benthic, littoral and pelagic prey, it is possible that Arctic char fed more in surface waters during their summer movements (Harris et al. 2020), in contrast to more benthic feeding of Greenland cod (Nielsen and Andersen 2001). In the benthos, Greenland cod would be exposed to colder and more consistent temperatures than Arctic char if they remained in shallower waters, which could influence metabolism and stable isotope fractionation rates (Barnes et al. 2007).

### Flexibility to climate change effects

Changes to conventional prey pulses, competitors (including sub-Arctic salmonids and gadids), and predators throughout the Arctic are altering the stage of ontogenetic trophic shifts for these species (Babaluk et al. 2000; Fossheim et al. 2015; Barbeaux and Hollowed 2018; Pettitt-Wade et al. 2020). Our findings highlight the potential benefits of shifting diets and habitat use leading to higher body condition (phase angle—tissue cell density). Increasing water temperatures can reduce body size and size-at maturity in fishes due to physiological constraints (Sheridan and Bickford 2011), which could enhance opportunities for density-dependent individual specialization (e.g., Svanbäck and Persson 2004; Svanbäck and Bolnick 2007) and diet-related habitat shifts (Schindler et al. 1997; Laurel et al. 2004). For example, stable isotope analysis of Arctic char in Cumberland Sound, Nunavut, indicated a dietary switch, from primarily amphipod crustaceans in 2004 to capelin in 2011 (Ulrich and Tallman 2021). Similarly, dietary shifts in Arctic char are reported at other geographic locations (e.g., Beaufort Sea: Harwood et al. 2015; Northern Labrador: Dempson et al. 2002). In contrast to Greenland cod, genetically synonymous Pacific cod exhibits high size-based individual specialization in long distance feeding migrations associated with their primary prey, walleye pollock, and their migrations are stretching towards higher latitudes (Laurel et al. 2009; Marsh et al. 2012; Westrheim 1996). Our findings provide a foundation for understanding how these gadids are responding to changes and further support the application of split-blood isotopes as a non-lethal measure of intraspecific variation in trophic and habitat shifts (Matich et al. 2019). Further research on other species using this analysis framework, combined with complementary data on feeding rates, metabolic efficiency, and movement patterns under different environmental conditions would further aid interpretation of these trends, and the trade-offs associated with contrasting patterns of intraspecific variation in trophic and habitat shifts.

## Conclusion

Our findings demonstrated that intraspecific variability in trophic shifts increases with body length for an anadromous salmonid in the western Canadian Arctic, yet there was no clear relationship for a perennially demersal marine gadid. Predictable size-based trends in intraspecific trophic variation of Arctic char highlight the importance of ontogeny for responding to ecosystem change through mobility. Conversely, lack of predictability in Greenland cod signifies an alternative generalist strategy that may promote high intraspecific variation across life stages, but may be dependent on consistent local prey diversity and available habitat. Arctic char and Greenland cod are both important for Inuit nutrition, culture and economy (Nuttall et al. 2005; Lea et al. 2023b), underscoring the value of understanding species-specific strategies to seasonal dynamicity and local prey and habitat availability that will have implications for Indigenous climate change adaptation approaches.

## Supplementary Information

Below is the link to the electronic supplementary material.

Supplementary file1 (PDF 499 KB)

## Data Availability

The data from this study is stored in an online repository operated by Fisheries and Oceans Canada and will be made available from the corresponding author on reasonable request. Metadata is available on the Polar Data Catalogue (CCIN 13212; https://www.polardata.ca/pdcsearch/).

## Code availability

SPSS syntax is available from the corresponding author upon reasonable request.

## References

[CR1] Andersson J, Byström P, Persson L, De Roos AM (2005). Plastic resource polymorphism: effects of resource availability on Arctic char (*Salvelinus alpinus*) morphology. Biol J Linn Soc.

[CR2] Babaluk JA, Reist JD, Johnson JD, Johnson L (2000). First records of sockeye (*Oncorhynchus nerka*) and pink salmon (*O. gorbuscha*) from Banks Island and other records of Pacific salmon in Northwest Territories, Canada. Arctic.

[CR3] Barbeaux SJ, Hollowed AB (2018). Ontogeny matters: climate variability and effects on fish distribution in the eastern Bering Sea. Fish Oceanogr.

[CR4] Barnes C, Sweeting CJ, Jennings S, Barry JT, Polunin (2007). Effect of temperature and ration size on carbon and nitrogen stable isotope trophic fractionation. Funct Ecol.

[CR5] Bearhop S, Adams CE, Waldron S, Fuller RA, MacLeod H (2004). Determining trophic niche width: a novel approach using stable isotope analysis. J Anim Ecol.

[CR6] Bolnick DI, Svanbäck R, Fordyce JA, Yang LH, Davis JM, Hulsey CD, Forister ML (2003). The ecology of individuals: incidence and implications of individual specialization. Am Nat.

[CR7] Bolnick DI, Amarasekare P, Araújo MS, Bürger R, Levine JM, Novak M, Rudolf VHW, Schreiber SJ, Urban MC, Vasseur DA (2011). Why intraspecific trait variation matters in community ecology. Trends Ecol Evol.

[CR8] Brewster JD, Giraldo C, Choy ES, MacPhee SA, Hoover C, Lynn B, McNicholl DG, Majewski B, Rosenberg B, Power M, Reist JD, Loseto LL (2018). A comparison of the trophic ecology of Beaufort Sea Gadidae using fatty acids and stable isotopes. Polar Biol.

[CR101] Burke TG, Pettitt-Wade H, Hollins JP, Gallagher C, Lea E, Loseto L, Hussey NE (2022). Evidence for three morphotypes among anadromous Arctic char (*Salvelinus alpinus*) sampled in the marine environment. J Fish Biol.

[CR9] Cox KW, Heintz R (2009). Electrical phase angle as a new method to measure fish condition. Fish B-NOAA.

[CR10] Dempson JB, Shears M, Bloom M, Magnan P, Audet C, Glémet H, Legault M, Rodríguez MA, Taylor EB (2002). Spatial and temporal variability in the diet of anadromous Arctic charr, *Salvelinus alpinus*, in northern Labrador. Ecology, behaviour and conservation of the charrs, genus *Salvelinus*.

[CR11] Dey CJ, Yurkowski DJ, Schuster R, Shiffman DS, Bittick SJ (2018). Patterns of uncertainty in life-history and extinction risk for Arctic vertebrates. Arct Sci.

[CR12] Dill LM (1983). Adaptive flexibility in the foraging behavior of fishes. Can J Fish Aquat Sci.

[CR13] Eloranta AP, Siwertsson A, Knudsen R, Amundsen PA (2011). Dietary plasticity of Arctic charr (*Salvelinus alpinus*) facilitates coexistence with competitively superior European whitefish (*Coregonus lavaretus*). Ecol Freshw Fish.

[CR14] Emmerton CA, Lesack LF, Vincent WF (2008). Nutrient and organic matter patterns across the Mackenzie River estuary and shelf during the seasonal recession of sea-ice. J Mar Syst.

[CR15] Fisher JA, Frank KT, Leggett WC (2010). Global variation in marine fish body size and its role in biodiversity–ecosystem functioning. Mar Ecol Prog Ser.

[CR16] Fokkema W, van der Jeugd HP, Lameris TK, Dokter AM, Ebbinge BS, de Roos AM, Nolet BA, Piersma T, Olff H (2020). Ontogenetic niche shifts as a driver of seasonal migration. Oecologia.

[CR17] Fossheim M, Primicerio R, Johannesen E, Ingvaldsen RB, Aschan MM, Dolgov AV (2015). Recent warming leads to a rapid borealization of fish communities in the Arctic. Nat Clim Change.

[CR18] Guiguer KRRA, Reist JD, Power M, Babaluk JA (2005). Using stable isotopes to confirm the trophic ecology of Arctic charr morphotypes from Lake Hazen, Nunavut, Canada. J Fish Biol.

[CR19] Hammer LJ, Hussey NE, Marcoux M, Pettitt-Wade H, Hedges K, Tallman R, Furey NB (2021). Arctic char enter the marine environment before annual ice breakup in the high Arctic. Environ Biol Fish.

[CR20] Hammer LJ, Hussey NE, Marcoux M, Pettitt-Wade H, Hedges K, Tallman R, Furey NB (2022). Arctic char (*Salvelinus alpinus*) movement dynamics relative to ice breakup in a high Arctic embayment. Mar Ecol Prog Ser.

[CR21] Hansen JH, Hedeholm RB, Sünksen K, Christensen JT, Grønkjær P (2012). Spatial variability of carbon (δ^13^C) and nitrogen (δ^15^N) stable isotope ratios in an Arctic marine food web. Mar Ecol Prog Ser.

[CR22] Harris LN, Yurkowski DJ, Gilbert MJ, Else BG, Duke PJ, Ahmed MM, Tallman RF, Fisk AT, Moore J (2020). Depth and temperature preference of anadromous Arctic char *Salvelinus alpinus* in the Kitikmeot Sea, a shallow and low-salinity area of the Canadian Arctic. Mar Ecol Prog Ser.

[CR23] Hartman KJ, Margraf FJ, Hafs AW, Cox MK (2015). Bioelectrical impedance analysis: a new tool for assessing fish condition. Fisheries.

[CR24] Harwood LA, Sandstrom SJ, Papst MH, Melling H (2013) Kuujjua River Arctic Char: monitoring stock trends using catches from an under-ice subsistence fishery, Victoria Island, Northwest Territories, Canada, 1991–2009. Arctic 66: 291–300 https://www.jstor.org/stable/23594631

[CR25] Harwood LA, Smith TG, George JC, Sandstrom SJ, Walkusz W, Divoky GJ (2015). Change in the Beaufort Sea ecosystem: diverging trends in body condition and/or production in five marine vertebrate species. Prog Oceanogr.

[CR26] Hobson KA, Ambrose WG, Renaud PE (1995). Sources of primary production, benthic-pelagic coupling, and trophic relationships within the Northeast Water Polynya: insights from δ^13^C and δ^15^N analysis. Mar Ecol Prog Ser.

[CR27] Hollins J, Pettitt-Wade H, Gallagher CP, Lea EV, Loseto LL, Hussey NE (2022). Distinct freshwater migratory pathways in Arctic char (*Salvelinus alpinus*) coincide with separate patterns of marine spatial habitat-use across a large coastal landscape. Can J Fish Aquat.

[CR28] Jackson JBC, Kirby MX, Wolfgang HB, Bjorndal KA, Botsford LW, Bourque BJ, Bradbury RH, Cooke R, Erlandson J, Estes JA, Hughes TP, Kidwell S, Lange CB, Lenihan HS, Pandolfi JM, Peterson CH, Steneck RS, Tegner MJ, Warner RR (2001). Historical overfishing and the recent collapse of coastal ecosystems. Science.

[CR29] Jørgensen EH, Johnsen HK (2014). Rhythmic life of the Arctic charr: adaptations to life at the edge. Mar Genom.

[CR30] Kahilainen KK, Thomas SM, Keva O, Hayden B, Knudsen R, Eloranta AP, Tuohiluoto K, Amundsen P-A, Malinen T, Järvinen A (2016). Seasonal dietary shift to zooplankton influences stable isotope ratios and total mercury concentrations in Arctic charr (Salvelinus alpinus (L.)). Hydrobiologia.

[CR31] Knickle DC, Rose GA (2014). Dietary niche partitioning in sympatric gadid species in coastal Newfoundland: evidence from stomachs and CN isotopes. Environ Biol Fish.

[CR32] Knickle DC, Rose GA (2014). Examination of fine-scale spatial-temporal overlap and segregation between two closely related congeners *Gadus morhua* and *Gadus ogac* in coastal Newfoundland. J Fish Biol.

[CR33] Knudsen R, Amundsen P-A, Primicerio R, Klemetsen A, Sørensen P (2007). Contrasting niche-based variation in trophic morphology within Arctic charr populations. Evol Ecol Res.

[CR34] Laurel BJ, Gregory RS, Brown JA (2003). Settlement and distribution of Age-0 juvenile cod, *Gadus morhua* and *G. ogac*, following a large-scale habitat manipulation. Mar Ecol Prog Ser.

[CR35] Laurel BJ, Gregory RS, Brown JA, Hancock JK, Schneider DC (2004). Behavioural consequences of density-dependent habitat use in juvenile cod *Gadus morhua* and *G. ogac*: the role of movement and aggregation. Mar Ecol Prog Ser.

[CR36] Laurel BJ, Ryer CH, Knoth B, Stoner AW (2009). Temporal and ontogenetic shifts in habitat use of juvenile Pacific cod (*Gadus macrocephalus*). J Exp Mar Biol Ecol.

[CR37] Lea EV, Gallagher CP, Carder GM, Matari KGA, Harwood LA (2023a) Ulukhaktok, Northwest Territories coastal Arctic Char (*Salvelinus alpinus*) subsistence (1993–1997 and 2011–2015) and commercial (2010–2015) fisheries: Catch-per-unit-effort and biological sampling. DFO Can Sci Advis Sec Res Doc 2023a/015. iv + 41 p. https://www.dfo-mpo.gc.ca/csas-sccs/publications/resdocs-docrech/2023/2023_015-eng.pdf

[CR38] Lea EV, Olokhaktomiut Hunters and Trappers Committee, and Harwood LA (2023b). Fish and marine mammals harvested near Ulukhaktok, Northwest Territories, with a focus on anadromous Arctic char (*Salvelinus alpinus*). DFO Can Sci Advis Sec Res Doc 2023b/014. iv + 23 p. https://publications.gc.ca/collections/collection_2023b/mpo-dfo/fs70-5/Fs70-5-2023b-014-eng.pdf

[CR39] Marsh JM, Hillgruber N, Foy RJ (2012). Temporal and ontogenetic variability in trophic role of four groundfish species—Walleye Pollock, Pacific Cod, Arrowtooth Flounder, and Pacific Halibut—around Kodiak Island in the Gulf of Alaska. Trans Am Fish.

[CR40] Matich P, Kiszka JJ, Heithaus MR, Le Bourg B, Mourier J (2019). Inter-individual differences in ontogenetic trophic shifts among three marine predators. Oecologia.

[CR100] McCarthy ID, Fraser D, Waldron S, Adams CE (2004). A stable isotope analysis of trophic polymorphism among Arctic charr from Loch Ericht, Scotland. J Fish Biol.

[CR41] McGovern M, Pavlov AK, Deininger A, Granskog MA, Leu E, Søreide JE, Poste AE (2020). Terrestrial inputs drive seasonality in organic matter and nutrient biogeochemistry in a high Arctic fjord system (Isfjorden, Svalbard). Front Mar Sci.

[CR42] Meager JJ, Fernö A, Skjæraasen JE (2018). The behavioural diversity of Atlantic cod: insights into variability within and between individuals. Rev Fish Biol Fish.

[CR43] Mikhail MY, Welch HE (1989). Biology of Greenland cod, *Gadus ogac*, at Saqvaqjuac, northwest coast of Hudson Bay. Environ Biol Fish.

[CR44] Moore JS, Harris LN, Kessel ST, Bernatchez L, Tallman RF, Fisk AT (2016). Preference for nearshore and estuarine habitats in anadromous Arctic char (*Salvelinus alpinus*) from the Canadian high Arctic (Victoria Island, Nunavut) revealed by acoustic telemetry. Can J Fish Aquat Sci.

[CR45] Morin B, Hudon C, Whoriskey F (1991). Seasonal distribution, abundance, and life-history traits of Greenland cod, *Gadus ogac*, at Wemindji, eastern James Bay. Can J Zool.

[CR46] Nielsen JR, Andersen M (2001). Feeding habits and density patterns of Greenland cod, *Gadus ogac* (Richardson 1836), at west Greenland compared to those of the coexisting cod, *Gadus morhua* L. J Northwest Atl Fish Sci.

[CR47] Nuttall M, Berkes F, Forbes B, Kofinas G, Vlassova T, Wenzel G, Berner J, Symon C, Arris L, Heal OW (2005). Hunting, herding, fishing and gathering: indigenous peoples and renewable resource use in the Arctic. Arctic climate impact assessment.

[CR48] Pettitt-Wade H, Pearce T, Kuptana D, Gallagher CP, Scharffenberg K, Lea EV, Hussey NE, Loseto LL (2020). Inuit observations of a Tunicata bloom unusual for the Amundsen Gulf, western Canadian Arctic. Arct Sci.

[CR49] Polis GA (1984). Age structure component of niche width and intraspecific resource partitioning: can age groups function as ecological species?. Am Nat.

[CR50] Rikardsen AH, Amundsen PA, Bjørn PA, Johansen M (2000). Comparison of growth, diet and food consumption of sea-run and lake-dwelling Arctic charr. J Fish Biol.

[CR51] Scarponi P, Coro G, Pagano P (2018). A collection of Aquamaps native layers in NetCDF format. Data Br.

[CR52] Schindler DE, Hodgson JR, Kitchell JF (1997). Density-dependent changes in individual foraging specialization of largemouth bass. Oecologia.

[CR53] Shapiera M, Gregory RS, Morris CJ, Pennell CJ, Snelgrove PV (2014). Season and site fidelity determine home range of dispersing and resident juvenile Greenland cod *Gadus ogac* in a Newfoundland fjord. Mar Ecol Prog Ser.

[CR54] Sheaves M (2009). Consequences of ecological connectivity: the coastal ecosystem mosaic. Mar Ecol Prog Ser.

[CR55] Sheridan JA, Bickford D (2011). Shrinking body size as an ecological response to climate change. Nat Clim Change.

[CR56] Shipley ON, Matich P (2020). Studying animal niches using bulk stable isotope ratios: an updated synthesis. Oecologia.

[CR57] Skúlason S, Parsons KJ, Svanbäck R, Räsänen K, Ferguson MM, Adams CE, Amundsen P-A, Bartels P, Bean CW, Boughman JW, England G, Guðbrandsson J, Hooker OE, Hudsen AG, Kahilainen KK, Knudsen R, Kristjánsson BK, Leblanc CA-L, Jónsson Z, Öhlund G, Smith C, Snorrason SS (2019). A way forward with eco evo devo: an extended theory of resource polymorphism with postglacial fishes as model systems. Biol.

[CR58] Spares AD, Stokesbury MJW, O’Dor RK, Dick TA (2012). Temperature, salinity and prey availability shape the marine migration of Arctic char, *Salvelinus alpinus*, in a macrotidal estuary. Mar Biol.

[CR59] Steiner N, Azetsu-Scott K, Hamilton J, Hedges K, Hu X, Janjua MY, Lavoie D, Loder J, Melling H, Merzouk A, Perrie W, Peterson I, Scarratt M, Sou T, Tallmann R (2015). Observed trends and climate projections affecting marine ecosystems in the Canadian Arctic. Environ Rev.

[CR60] Stroeve JC, Serreze MC, Holland MM, Kay JE, Malanik J, Barrett AP (2012). The Arctic’s rapidly shrinking sea ice cover: a research synthesis. Clim Change.

[CR61] Svanbäck R, Bolnick DI (2007). Intraspecific competition drives increased resource use diversity within a natural population. Proc R Soc B.

[CR62] Svanbäck R, Persson L (2004). Individual diet specialization, niche width and population dynamics: implications for trophic polymorphisms. J Anim Ecol.

[CR63] Ulrich KL, Tallman RF (2021). Multi-indicator evidence for habitat use and trophic strategy segregation of two sympatric forms of Arctic char from the Cumberland Sound region of Nunavut, Canada. Arct Sci.

[CR64] Vander Zanden MJ, Cabana G, Rasmussen JB (1997). Comparing trophic position of freshwater fish calculated using stable nitrogen isotope ratios (δ^15^N) and literature dietary data. Can J Fish Aquat Sci.

[CR65] Vander Zanden MJ, Clayton MK, Moody EK, Solomon CT, Weidel BC (2015). Stable isotope turnover and half-life in animal tissues: a literature synthesis. PloS One.

[CR66] Werner EE, Gilliam JF (1984). The ontogenetic niche and species interactions in size-structured populations. Ann Rev Ecol Syst.

[CR67] Westrheim SJ (1996). On the Pacific cod (Gadus macrocephalus) in British Columbia waters, and a comparison with Pacific cod elsewhere, and Atlantic cod (*G. morhua*). Can Tech Rep Fish Aqua Sci.

[CR68] Yurkowski DJ, Hussey NE, Ferguson SH, Fisk AT (2018). A temporal shift in trophic diversity among a predator assemblage in a warming Arctic. R Soc Open Sci.

