# Supplementary material for: Contrasting intra-individual variation in size-based trophic and habitat shifts for two coastal Arctic fish species
Source: Oecologia. 2023 Jul 24;202(3):601–16. doi: 10.1007/s00442-023-05423-9 (PMC10386975; doi:10.1007/s00442-023-05423-9)

Electronic Supplemental Material (ESM) Online Resources

Contrasting intra-individual variation in size-based trophic and habitat shifts for two coastal Arctic fish species

Harri Pettitt-Wade[1,2,*], Nigel E. Hussey[2], Colin P. Gallagher[1], Ellen V. Lea[3], Danielle L. Orrell[2], Lisa L. Loseto[1,4]

[1]Fisheries and Oceans Canada, Freshwater Institute, Winnipeg, MB. Canada. R3T 2N6
[2]Integrative Biology, University of Windsor, Windsor, ON. Canada. N9B 3P4
[3]Fisheries and Oceans Canada, Inuvik, NT. Canada. X0E 0T0
[4]Environment and Geography, University of Manitoba, Winnipeg, MB. Canada. R3T2N2

Author contributions: NEH, HPW and LLL conceived the study design. HPW, DLO, CPG and EVL conducted the fieldwork. HPW conducted laboratory work and analysed the data with detailed input from NEH and LLL. HPW, NEH and LLL wrote the manuscript with detailed input during drafts from CPG, EVL and DLO. All authors provided editorial advice prior to submission.

Running head: Intra-individual variation in size-based trophic and habitat shifts

*Correspondence: pettitth@uwindsor.ca

Online Resource 1 – Interpreting regression results

Figure 1 Conceptual two-tissue stable isotope residual – body size plots with description adapted from Matich et al. (2019)

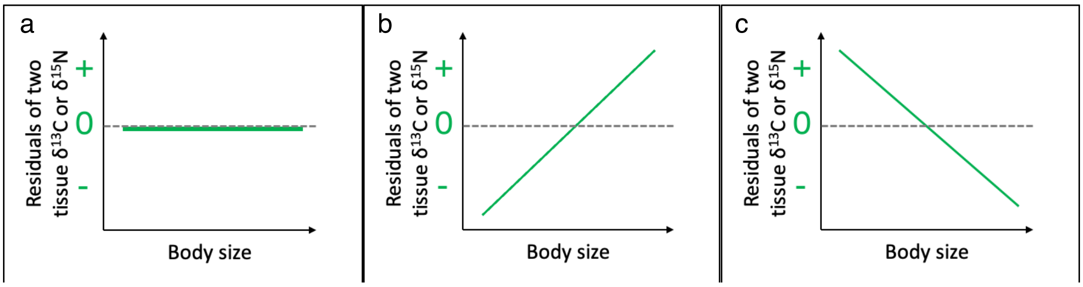

(a) Non-significant slope (i.e., no relationship) indicates consistent magnitude and direction in $\delta^{13}C/\delta^{15}N$ shifts, or no shift in trophic interactions (equal fast and slow turnover). (b) Positive slope indicates faster enrichment in 13C/15N of fast turnover tissue compared to slow turnover tissue with body size, indicative of increased consumption of 13C-/15N-enriched food with ontogeny. (c) Negative slope indicates faster depletion in 15N of the fast turnover tissue compared to the slow turn-over tissue with body size, indicative of increased consumption of 13C-/15N-depleted food with ontogeny.

Online Resource 2 – Stable isotope δ13C and δ15N bi-plots

Figure 1 Paired tissue linear regressions of (a) δ13C and (b) δ15N in plasma and red blood cells (RBC) from Arctic char and Greenland cod.

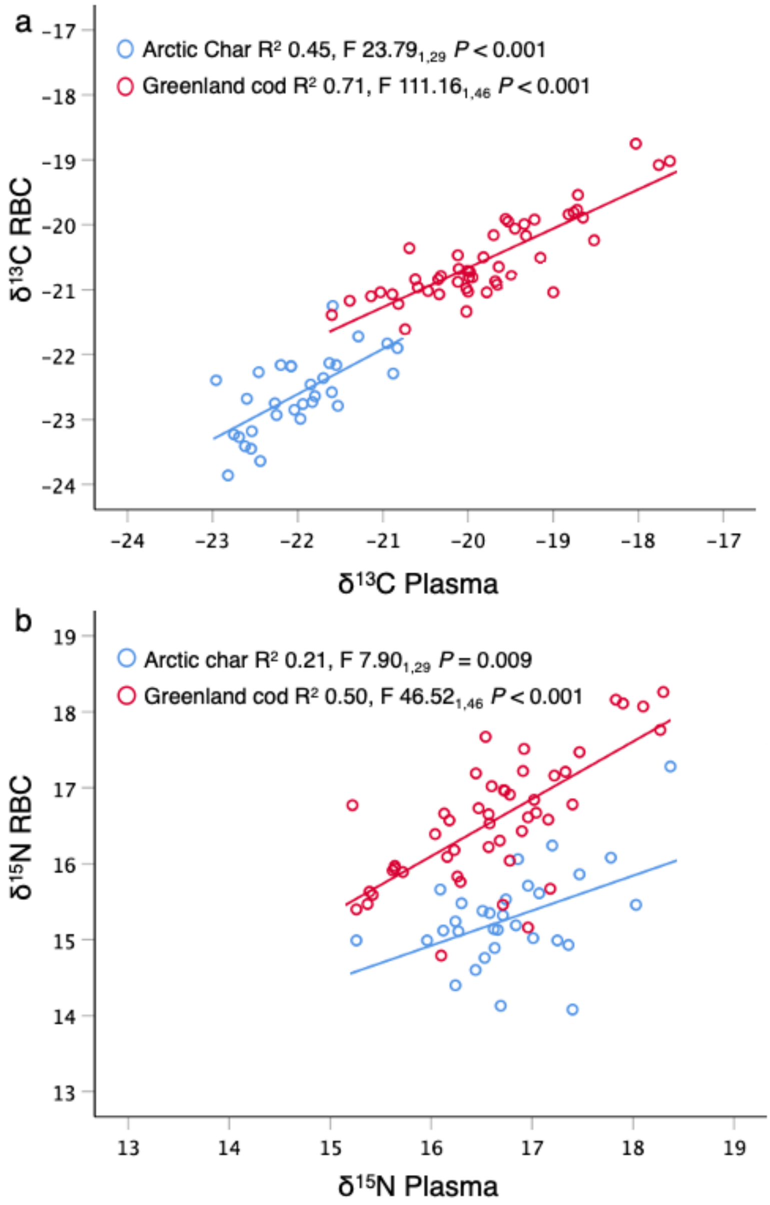

Online Resource 3 – Regressions with body condition (phase angle)

Figure 1 Linear regressions of body length against phase angle (a, b) and phase angle against absolute $\delta^{15}N$ tissue difference body length residuals (c, d) in Arctic char and Greenland cod. A diet-tissue correction was applied prior to calculating tissue differences.

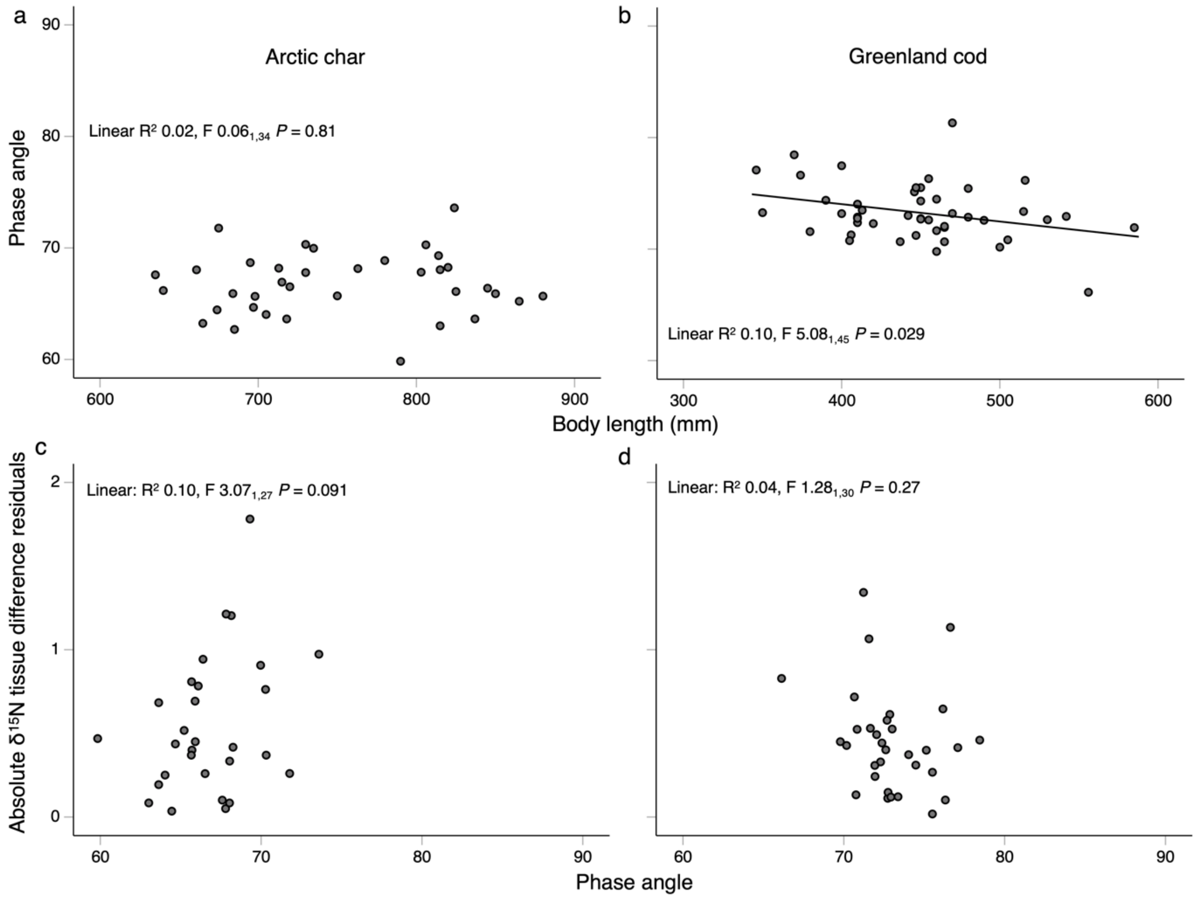

Figure 2 Linear regressions of phase angle against absolute δ^{13}C body length residuals from plasma and red blood cell (RBC) in Arctic char (a) and Greenland cod (b). Body length is fork length in Arctic char, total length in Greenland cod (mm)

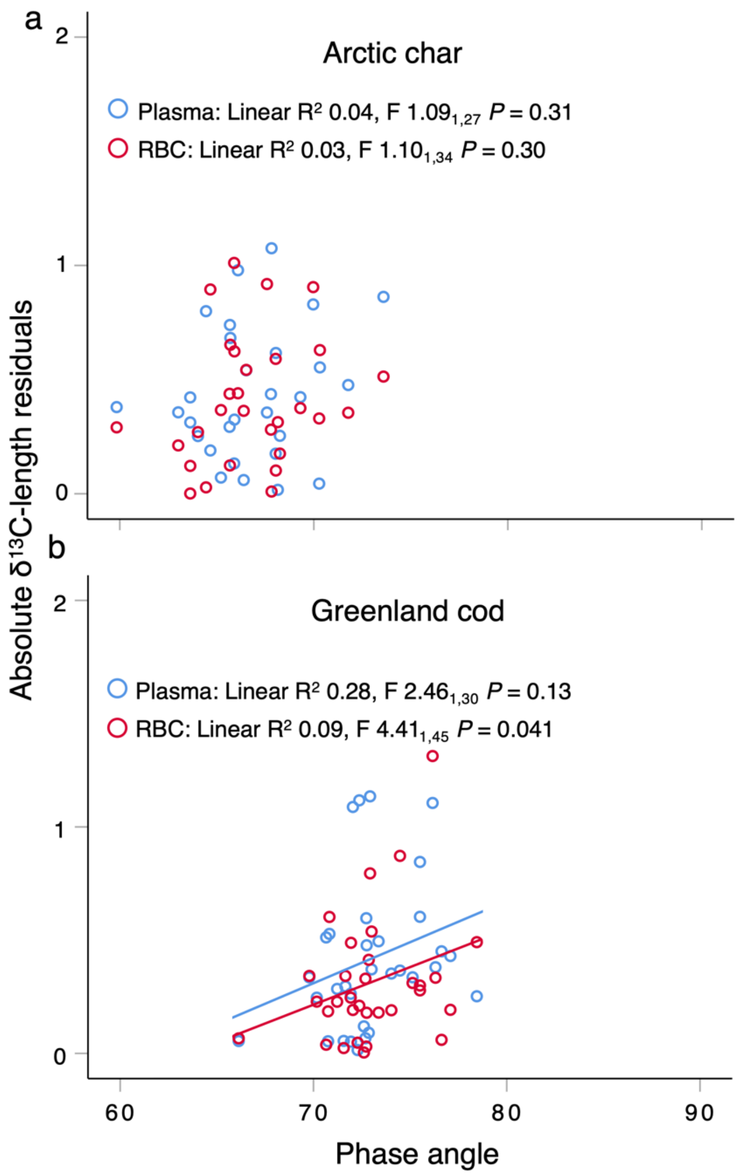

Supplement: Supplementary file 1 — Supplementary file1 (PDF 499 KB) [file 442_2023_5423_MOESM1_ESM.pdf]
